# Lay knowledge of cervical cancer in Manhiça district, Mozambique: a qualitative study

**DOI:** 10.1186/s12978-020-00980-1

**Published:** 2020-08-24

**Authors:** Yara Alonso Menendez, Olga Cambaco, Carolina Mindú, Hoticha Nhantumbo, Titos Uamusse, Graça Matsinhe, Benigna Matsinhe, Rosa Marlene Manjate, Azucena Bardají, Clara Menendez, Esperança Sevene, Khátia Munguambe

**Affiliations:** 1 Manhiça Health Research Centre (CISM), Rua 12 - Cambeve, Manhiça, Mozambique; 2 Extended Program on Immunization (PAV), Ministry of Health (MISAU), Maputo, Mozambique; 3 National Directorate for Public Health (DNSP), Ministry of Health (MISAU), Maputo, Mozambique; 4 Barcelona Institute for Global Health (ISGLOBAL)/Hospital Clinic-Universitat de Barcelona, Barcelona, Spain; 5 Faculty of Medicine, Eduardo Mondlane University (UEM), Maputo, Mozambique

**Keywords:** Cervical cancer, Sexual and reproductive health, Lay perceptions, Mozambique

## Abstract

**Background:**

Mozambique has one of the highest cervical cancer incidence rates in the world. Health interventions are still being conceived solely from a non-communicable disease standpoint despite that it is also a sexual and reproductive health problem. The objective of this study was to assess the extent to which lay perceptions of cervical cancer align with biomedical knowledge from the standpoint of sexual and reproductive health.

**Methods:**

10 focus group discussions were carried out with 10 target groups in Manhiça. The target groups were diverse in terms of age, sex, educational level and occupation. There were a total of 116 participants. The focus groups discussions were applied to obtain verbal information and trigger debates around beliefs and attitudes about cervical cancer as well as to explore notions of transmission and aetiology of the disease. The discussions were recorded for later transcription and analysis, following a combination of content and thematic analysis.

**Results:**

Participants were familiar with the biomedical term ‘cervical cancer’ but knowledge of its aetiology and transmission was limited. Cervical cancer was readily associated to sexual transmission and sexually transmitted infections, and conceived as a ‘wound that does not heal’. The term ‘cancer’ caused confusion, as it was perceived to happen only in limbs, understood as hereditary, not transmissible and as an illness of the West.

**Conclusions:**

Lay perceptions of cervical cancer do, to a large extent, align with biomedical ones, thus, there is common ground to frame future health interventions from a sexual and reproductive health standpoint. Some misperceptions were identified which could be reduced through social behaviour change communication initiatives.

## Resumo em Português

**Antecedentes:**

Moçambique tem uma das maiores taxas de incidência de cancro do colo do útero no mundo. As intervenções de saúde para o cancro do colo do útero ainda têm sido concebidas apenas do ponto de vista de doenças não transmissíveis, apesar do cancro do colo do útero ser também uma problema de saúde sexual e reprodutiva. O objectivo do estudo foi de avaliar em que medida as percepções leigas sobre o cancro do colo do útero alinham-se com o conhecimento biomédico do ponto de vista de saúde sexual e reprodutiva.

**Métodos:**

Foram realizadas 10 discussões em grupos focais com 10 grupos-alvo no distrito de Manhiça. Os grupos-alvo eram diversos em termos de idade, sexo, nível de educação e ocupação. O estudo envolveu um total 116 participantes. As discussões em grupos focais geraram informação verbal e desencadearam debates sobre crenças e atitudes em relação ao cancro do colo do útero, e também exploraram noções de transmissão e etiologia da doença. As discussões foram gravadas para posterior transcrição e análise, seguindo uma combinação de análise de conteúdo e temática.

**Resultados:**

Os participantes estavam familiarizados com o termo biomédico “cancro do colo do útero “, mas o seu conhecimento sobre a sua etiologia e transmissão era limitado. O cancro do colo do útero foi associado à transmissão sexual e infecções sexualmente transmissíveis e concebido como uma “ferida que não cicatriza”. O termo “cancro” causou confusão, pois era percebido como ocorrendo apenas nos membros do corpo, entendido como hereditário, não transmissível e como uma doença do Ocidente.

**Conclusão:**

As percepções leigas sobre o cancro do colo do útero alinham-se, em grande parte às definições biomédicas, portanto, há uma estrutura de base comum para enquadrar futuras intervenções de saúde do ponto de vista da saúde sexual e reprodutiva.

## Plain English summary

Cervical cancer is the 4th most common cancer among women globally, although 85% of the deaths happen in low and middle-income countries, like Mozambique. Cervical cancer is the most frequent cancer among women and approximately 4000 women die every year from the disease. Most cancers are not transmissible and so prevention is addressed differently. Cervical cancer is a sexual and reproductive health issue, mainly because 99% of cases are associated to infection by HPV, a sexually transmitted virus. In Mozambique, cervical cancer health interventions are still being designed in the same way as other cancers that are not transmissible so the goal of this study was to examine how people understand this disease and whether their perceptions agreed with the sexual health medical perspective. We did this by conducting 10 focus group discussions with different groups of participants, accounting for differences in age, sex, educational level and occupation. The study took place in Manhiça, a rural district in Southern Mozambique. We found that participants were familiar with the term cervical cancer but had limited knowledge of its definition and causes. We also found it was often associated with sexual transmission and understood as a ‘wound that doesn’t heal’. At the same time, it was also perceived to be hereditary and cancer was thought to be something that did not happen in the womb. Our conclusion is that there is some overlap between the medical and popular notions of cervical cancer as a sexual health issue that should be exploited to make health interventions more effective.

## Introduction

Cervical Cancer (CC) is the 4th most common cancer among women globally. There are 528,000 new cases and 266,000 deaths per year due to CC worldwide [1]. Ninety-nine per cent of CC cases are associated to Human Papilloma Virus (HPV) infection [2]. Persistent infection with specific types of HPV (most commonly types 16 and 18) can lead to precancerous lesions of the cervix, which if untreated, may result in CC after several years [3].

Effective screening programs can significantly reduce CC incidence and mortality if they succeed in ensuring early detection of pre-cancerous lesions [3]. This has been achieved in most high-income countries, which has not been the case for low and middle income countries (LMICs), principally due to these countries’ lack of appropriate infrastructure, equipment and human resources for screening and treatment [4–6]. As a result, there is a disproportional distribution of CC incidence and mortality between high and LMICs. The latter carry the burden of 85% of deaths from CC [1].

Mozambique ranks amongst the top 20 countries with highest CC rates in 2018 [7] and approximately 8 million women aged ≥15 years are at lifetime risk of developing CC [8], making this the most frequent cancer among Mozambican women [9]. The country’s crude CC incidence rate is 42–60/100,000 women/year [8, 9], one of the highest in the world. Every year 5622 women are diagnosed with CC and 4061 die from the disease [8]. In 2009, the Mozambican Ministry of Health (MoH) launched the CC screening program based on visual inspection with acetic acid (VIA) and cryotherapy in selected health facilities [5, 10]. Both the rollout of the screening program and the countrywide introduction of HPV vaccination require an in-depth examination of the local perceptions regarding CC in order to best frame the interventions.

The key premise of this article is the idea that framing a health intervention around beliefs, interests and concerns of those involved, enables implementers to address the points of disagreement between theirs and the beneficiaries´ perspectives, increasing the likelihood of acceptability of the intervention, hence maximizing uptake and effectiveness [11–13].

To date, studies have reported mixed levels of awareness of CC and consistently low levels of knowledge of CC and HPV across Sub-Saharan Africa (SSA) [14]. Lay perceptions of CC in a number of LMICs reveal sexual connotations, some of which partially touch upon biomedical explanatory models [15]. This was notable with regards to perceived risk factors, with references to multiple sexual partners, exposure to sexually transmitted infections (STIs), early onset of sexual activity, poor genital hygiene and history of abortions [16–19].

From the biomedical perspective, there is no doubt that CC is a sexual and reproductive health concern for the following reasons. Firstly, sexual contact is the most common requisite for HPV transmission, the precursor of CC. Secondly, several risk factors for the development of CC are related to sexual and reproductive health, namely early sexual debut, multiple sexual partners, long term use of oral contraceptives, parity, and exposure to other STIs [20–22]. Thirdly, invasive CC is presented with several signs and symptoms which affect sexual and reproductive life in particular: vaginal discomfort, odorous discharge, irregular inter-menstrual vaginal bleeding, vaginal bleeding after sexual intercourse, to name just a few [3]. Finally, besides damage of the reproductive tract by cancerous lesions, the common forms of treatment may lead to sexual and bladder dysfunction, and radical surgery impairs fertility [23].

The preposition underpinning the proposed analysis is that interventions to control CC, which in Mozambique are still being conceived from a non-communicable disease (NCD) standpoint [24, 25], and specifically focused on cancer, are not guaranteed to gain a high buy-in from communities. This is because on one hand cancer is not yet an openly talked about issue, neither in Mozambique nor in other sub-Saharan African countries, and on the other hand it is subject to fear-based and fatalistic attitudes, discouraging people from timely seeking diagnosis and treatment [19, 26, 27]. Based on the already established reproductive health programs with reported progress in these settings, it is possible that if CC is also framed around a sexual and reproductive health point of view, communities are likely to face the problem with a different perspective than it would be if the highlight remains on the disease being one of many other types of cancer. The aim of this analysis is to assess the extent to which lay perceptions of CC in a rural area of southern Mozambique align with biomedical perspectives of CC from the standpoint of sexual and reproductive health.

## Methods

### Study site and population

The study took place in Manhiça, a rural, in-land district of Southern Mozambique, located 80 km away from Maputo City. The district covers an area of 2360 Km^2^*.* It has a population of 188, 405, of which 47,168 are women of reproductive age (15–49 years of age). The population is mostly Changana, the dominant ethnic group in Southern Mozambique, and Christianity is the main religion. The language spoken in this region is Changana, the language spoken by the ethnic majority, whilst Portuguese is the official language spoken across the country. For most people, Changana is the day-to-day language even though Portuguese is often used interchangeably. The district is served by reasonable roads, communication-infrastructure and, in comparison to similar rural districts in the country, relatively good access to health-care and education. Manhiça hosts one rural hospital, one district hospital and 17 peripheral health centres. This data was retrieved from Manhiça Health Research Centre’s (CISM) Health and Demographic Surveillance System [28].

### Study design

This study was nested within a broader mixed methods study that employed a survey questionnaire and a qualitative enquiry into perceptions of CC and acceptability of HPV vaccine, which took place from October 2013 to March 2014 in three districts in Mozambique, namely Mocímboa da Praia, Ka-Mavota and Manhiça [29]. The study was conducted to inform the HPV vaccine national demonstration programme which targeted 10-year old girls and took place in 2014 and 2015.

The present analysis focuses on the qualitative component of the study in the district of Manhiça. This study component, which draws from one data collection technique – the focus group discussion (FGD) – was conceptualized to examine the local knowledge and lay perceptions of CC (*emic* perspectives) and the extent to which they align or diverge from how this problem is defined from the point of view of biomedicine (the *etic* perspective) in order to infer on implications for the introduction of CC preventive strategies, as conceived by public health practitioners [30].

### Recruitment and data collection

Participants were recruited through a combination of purposive and snowball sampling. Recruitment was done via the *secretários do bairro*, who are community leaders at the level of the neighbourhood. FGD facilitators reached out to community leaders and briefed them about the inclusion criteria for each study target group. Community leaders consequently supported the FGD facilitators in the identification of up to 3 index participants per FGD, whom in turn helped identify further participants based on the inclusion criteria.

The target groups consisted of: adolescent girls and young women (aged 13 to 20), hereafter referred to as adolescent girls; older adolescents and youth (both male and female, aged 19 to 31), hereafter referred to as youth; caretakers (mostly parents or guardians of adolescents); community-based health workers (CHW); traditional healers; matrons (matrons are elderly women, though men can at times also be identified as such, who are respected and recognized in a given community as bearers of medicinal knowledge and sources of advice – not limited to health – but that are not traditional healers); unlicensed drug vendors (vendors selling drugs outside of the formal health system and without the required license but that are very common and constitute on additional health actor); community leaders; female elders and adult women of reproductive age (WRA). Most groups involved women and men, with a few exceptions: the group of WRA, the group with adolescents, the female elders and the matrons (with the exception of one male participant). Socio-demographic data from each participant was captured one-on-one after each FGD and registered in a designated form.

We constructed the target groups based on two criteria. Firstly, persons that due to their unique position have acquired specialized knowledge derived from assisting WRA (among others) and are likely to have encountered complaints potentially related to CC: matrons, traditional healers, unlicensed drug vendors, community leaders and CHWs. Secondly, persons belonging to natural groups organized on the basis of their lived experience and/or social role with regards to reproductive health who might themselves experience reproductive health problems or assist a relative or close acquaintance in care-seeking for a reproductive health problem: younger adolescents, older adolescents and youth, adult WRA, adolescents’ caretakers and female elders.

Ten FDGs were carried out (one per target group), each involving between 10 and 16 participants, with the exception of one group (unlicensed drug vendors), which consisted of only 3 participants. This group is generally hard to reach and, due to fear of repercussions, only 3 acceded to participate. Each FGD was guided by a facilitator and observed by one note-taker. Each FGD was conducted by a trained social science research assistant with a combination of medical and social science backgrounds to ensure a good balance between their capacity to engage in in-depth discussions on social behavioural aspects and being familiar with the biomedical implications of the topics of interest.

**Fig. 1:**
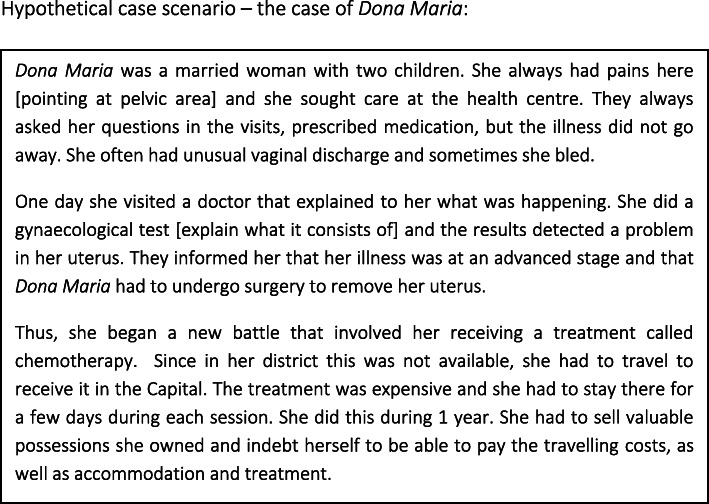
Hypothetical case scenario for cervical cancer

**Fig. 2:**
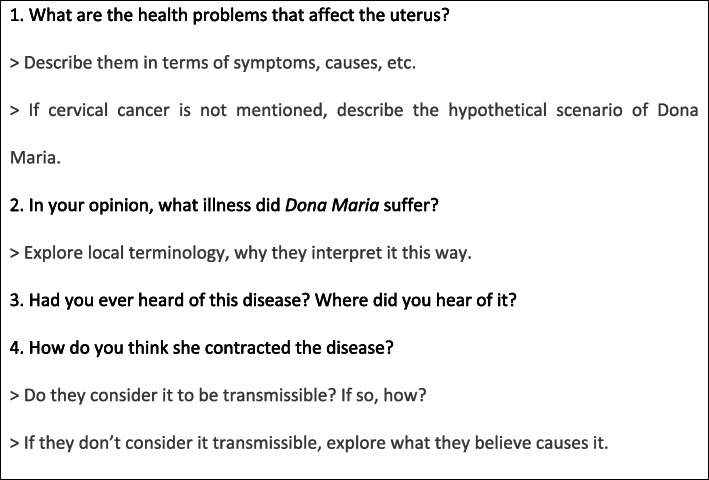
Focus Group discussion topic guide

FGDs were used to obtain verbal information and trigger debates around beliefs, attitudes and understandings of CC. Rather than initiating these discussions with the usual *etic* question about participants’ familiarity with the term “cervical cancer”, the sessions began with an open-ended question about the medical conditions that participants were familiar with in relation to the uterus, thus prompting them to describe all illnesses they could think of. This was followed by the presentation of a hypothetical case scenario (see Fig. 1), which had been developed by the study team in collaboration with clinicians, based on true stories of women suffering from invasive CC. FGD facilitators presented the scenario to participants and encouraged them to comment by probing them into the recognition of signs and symptoms suggestive of invasive CC and the extent to which they attribute such signs and symptoms to CC or other health problems. The facilitator posed follow-up questions based on a guide of open-ended questions (see Fig. 2) in order to gain further insights that would address the relevant research questions. Some sessions were conducted in the local language, Changana, and others conducted in Portuguese, depending on participants’ preference.

### Data management and analysis

With the permission of participants, the FGDs were captured by means of digital voice recorders Olympus AS-2400PC and transcribed verbatim using MS Word and, when necessary, simultaneously translated from Changana into Portuguese, with the exception of idioms and names that made reference to illnesses that had not evident equivalent in Portuguese. This was done in order to avoid distorting comparisons between emic and etic perspectives by conducting imprecise translations. Instead, original names were retained and were attached to a description of their meaning (in Portuguese). Whilst the facilitators transcribed and translated their own material, the team supervisor conducted quality checks consisting of reading the complete transcript whilst confronting the contents with the original recordings and feeding back to the research assistants responsible for the transcription for corrections.

A combination of content and thematic analysis was conducted by a social scientist, overseen by the principal investigator and supported by the fieldwork coordinator. Quality checked transcripts were then organized along thematic lines to aid analysis. For this analysis, a priori broad codes based on the research objectives were created to capture local names of potential illnesses linked to the signs and symptoms presented in the hypothetical case scenario, participants’ perceived causes of the identified illnesses that were compatible with CC and the potential link between CC and sexual practice. These codes were then revised and updated as new themes emerged during the data revision and immersion process. New data-driven themes emerged, particularly regarding local conceptions and misconceptions of CC and symptomatically compatible illnesses. All passages of texts were coded according to the relevant, a priori and data-driven codes.

### Ethical considerations

Prior to the FGDs, all participants received individual participant information sheets with the details of the nature of the study and implications for participation. Those willing to participate voluntarily signed a written informed consent form. If any name or description enabling identification of participants or third parties was brought up during the recorded FGDs, they were replaced with pseudonyms or generic adjectives in the act of transcription. Since the general objective of the study was to inform an intervention directly targeting girls as young as 10 years old, it was imperative to include under-aged participants. Thus, a written informed consent was requested from parents or guardians of participants under 18 years of age, and a written assent was obtained from the participants themselves.

## Results

### Participants’ characteristics

A total of 116 participants took part in the study. Ages varied greatly depending on the group but overall ranged from 12 to 80 years (more detailed information per group in Tables 1 and 2). Many of the participants were farmers (34%) and the majority had attained some level of primary education (63%). The overwhelming majority of participants were women (86%) given that many of the groups were purposely female-only.

**Table 1:** General socio-demographic description of participants (for detailed version see Table 2)

	TOTAL (116)
SEX*	
Female	86 (74%)
Male	14 (12%)
**AGE MEAN (+SD)**	38,4 (16,6)
**MARITAL STATUS**	
Single	46 (40%)
Married/marital union	58 (50%)
Widow	12 (10%)
**OCCUPATION**	
Student	8 (7%)
Farmer	39 (34%)
Domestic worker	18 (15%)
Worker other**	50 (43%)
Unemployed	1 (0,8%)
**EDUCATIONAL ATTAINMENT**	
Primary or less	83 (72%)
Secondary (complete or in part)	21 (18%)
None	12 (10%)

*Missing data on sex for 16 participants

**Includes traditional healers and community health workers

**Table 2:** Detailed socio-demographic characteristics of participants per target group

	GROUP										TOTAL (116)
	AG (13)	UV (3)	MAT (13)	WRA (16)	Y (11)	C-T (11)	FE (10)	CHW (16)	CL (12)	TH (11)	
**SEX**											
Female	13	2	12	16	5	9	10	*	9	10	86
Male	NA	1	1	NA	6	2	NA	*	3	1	14
**AGE MEAN (+SD)**	15.9 (3,4)	40 (7,8)	53,2 (9,3)	26,6 (9,5)	24 (3,4)	44 (14,9)	55,6 (9,6)	36,6 (11)	56 (9,5)	47,3 (11)	38,4 (16,6)
**MARITAL STATUS**											
Single	11	0	2	3	5	4	2	8	4	7	46
Married/marital union	2	3	6	13	6	7	2	8	8	3	58
Widow	0	0	5	0	0	0	6	0	0	1	12
**OCCUPATION**											
Student	8	0	0	0	0	0	0	NA	0	NA	8
Farmer	0	0	11	1	3	7	10	NA	7	NA	39
Domestic Worker	4	0	0	13	0	0	0	NA	1	NA	18
Employed other**	1	3	2	2	8	3	0	NA	4	NA	23
Unemployed	0	0	0	0	0	1	0	NA	0	NA	1
**EDUCATIONAL ATTAINMENT**											
Primary or less***	8	1	11	12	6	10	6	10	10	9	83
Secondary (complete or in part)	5	2	0	3	5	0	0	6	0	0	21
None	0	0	2	1	0	1	4	0	2	2	12

*Missing data

**Mostly working in commerce, but also in security and as ‘*chefe do quarteirão’* (head of a block of houses)

***Including adult education classes, commonly known as ‘*alfabetização’* (alphabetization), targeted for adults that never attended school with the objective of transmitting basic literacy skills

Abbreviations of target groups: Adolescent Girls (AG), Unlicensed Vendors (UV), Matrons (MAT), Women of Reproductive Age (WRA), Youth (Y), Care-takers (C-T), Female Elders (FE), Community Health Workers (CHW), Community Leaders (CL), Traditional Healers (TH)

### Recognition of the term “cervical cancer”

Participants in half of the groups that participated in FGDs introduced the term “cervical cancer” either when brainstorming about medical conditions affecting the uterus or after being presented with the hypothetical case scenario of the patient with advanced CC. The groups that brought forth the term were the community leaders, CHW, WRA, matrons and unlicensed drug vendors. None of the younger groups (youth and adolescent girls), as well as the caretakers, traditional healers and female elders introduced the term to the discussion, even after being presented with the scenario. When it became clear these participants were not relating the symptoms to CC, the facilitator would directly ask them whether they had heard of the term CC. Traditional healers, the youth and caretakers all claimed to have heard of *cancer* but were completely unaware of the existence of *cervical cancer*. Only the female elders and adolescent girls groups reported having heard of CC, following probing. In fact, various participants from the female elders group knew women that had died after having CC-compatible symptoms and their uterus removed, they were just not familiar with the biomedical term.

It must be noted that in many discussions during which the term was raised by participants, it was only one participant who did so and often without echoing from the rest of the group. For instance, in the case of the group of WRA, only one participant was familiar with CC due to the fact that her mother had suffered, and eventually died from, CC. Yet the rest of the group did not recognize the term or the symptoms she described as she retold the story of how the disease unfolded.

### Knowledge of cervical cancer

#### Perceived signs and symptoms

Amongst the groups that did introduce the term CC into the discussion without probing, there were varying degrees of biomedically-sound knowledge. In the case of community leaders, CC was described as an illness that causes symptoms such as ‘dirty’ vaginal discharge (*sujidade*), pelvic pains/cramps and difficulty in, and pain whilst, urinating.

*P – Yes, the uterus is always discharging dirt … It is like it is spilling … But it could also not be cancer, just because we think it is …*

(Participant, community leaders)

This participant indicates that symptoms such as discharge point at CC but could also be due to something else, thus hinting at the fact that CC symptoms are compatible with other medical conditions and hence easily confused. On the other hand, unlicensed drug vendors as well as CHWs also mentioned CC but did not have any knowledge of what it could consist of. They claimed they had only heard the name and knew that it is a deadly illness but knew nothing about its etiology.

*P – We only know, we only heard that... that that illness can kill. That one, the cervical cancer.*

(Participant, CHWs)

The participant whose mother had died of CC described it as causing a wound within her that would not heal, which in turn caused pelvic pain, weakness and affected her urine, making it gain a ‘milky’ appearance. The story she retold was very much in line with the hypothetical scenario that was presented at a later point:

*In the end it was that wound that stays in the uterus. She then went to the hospital … there they told her, they even did not tell her personally, they called us, her children, to tell us she had cancer. [ … ] They transferred her to the central hospital to remove her uterus but due to her tension they could not so they medicated her but she died a year later.*

(Participant, WRA group)

Even though they were amongst those that did not mention CC when brainstorming about uterine ailments, the group of matrons immediately remarked that the case scenario described had to be a case of CC. They justified the fact that they had not thought of it initially by explaining that they were not aware cancer could take place in the cervix/uterus since, to their knowledge, it could only affect other body parts. In consonance with the group of WRA, they conceived of cancer as an illness that caused ‘wounds that don’t heal’. Moreover, to their knowledge cancer was an illness of the West (of ‘white people’s countries’), uncommon in their environment and thus not a customary concern regarding reproductive health.

*P – No, we mean that we are not used to it because this illness is not common here in our country, it is more common in white people’s countries. So, we only heard this, that cancer attacks the legs, the arms or even the head, then they say that person has cancer because that wound does not heal. That it can also happen in the uterus or in the breasts, we did not know.*

(Participant, matrons)

Once the facilitator introduced the term CC to the discussion and clarified that the scenario was describing a case of CC, the participants of the FDG with female elders brought forth many cases they were familiar with that had gone through similar processes: pains during intercourse, weakness, cramps, foul-smelling discharge and intermittent vaginal bleeding. In these cases, the uterus was described to be ‘rotting’.

#### Conditions compatible with CC symptoms

When asked to interpret the scenario describing a typical case of advanced CC, those who did not mention CC were either unable to give any answer or provided names of other medical conditions that had compatible symptoms, such as *mudzoko, xicandzamete,* HIV, syphilis, myomas and STIs.

Participants in the TH’s FGD referred to the case scenario as *mudzoko*, a term in Changana that refers to a wound that does not cure. This is in line with the descriptions of CC symptoms previously mentioned. The term *xicandzamete* has been loosely translated as gonorrhea (even though at times it is used to refer to other STIs) and was the illness that was most commonly mentioned during the FDGs, either during the initial brainstorming about uterine ailments or as a plausible interpretation of the case scenario. Participants in both the adolescent girls’ group as well as the youth group concluded the case was referring to *xicandzamete*. However, in the youth group it was also considered the scenario could be a case of HIV.

The unlicensed drug vendor’s group claimed the scenario could refer to many STIs, particularly syphilis, whilst the group of matrons interpreted it as myomas. Remarkably, although participants in the group of female elders did not interpret the case to be CC, once they were explained what CC was they revealed there is a ‘traditional’ name (what seemed to imply ‘outdated’) in Changana that describes the exact same symptoms as CC: *xitsunhe.*

#### Knowledge about transmission

With regards to whether CC is transmissible and how it can be contracted, various themes emerged. In two groups (community leaders and drug vendors) there was some degree of consensus regarding the role of male circumcision. In their view, uncircumcised men are responsible for transmitting STIs to women because the ‘dirt’ (*sujidade)* and illnesses are stored in the foreskin which then enters the woman’s vagina (and eventually her uterus), causing infections, discharge and blisters which then evolve into cancer.

*P – I heard many times that it is we men that give this illness to our wives [ … ] when we don’t circumcise [ … ] That skin can also provoke that cancer illness.*

(Participants, unlicensed vendors)

Other groups were more divided with respect to whether CC could be transmitted at all. During the FGD with WRA most participants seemed to agree it was not transmissible in any way, whilst one person claimed it could be sexually transmitted. She argued that if a man and a woman have intercourse without protection and she is having the discharge caused by CC, the man would contract it and could then potentially transmit it to someone else. This discharge, she claimed, was what caused the wounds of the cancer. This description seemed to convince the rest, many of which eventually agreed with her.

Participants in the FDG with elderly women also had conflicting views; for a few of them CC could not be transmitted, whilst others claimed it could be sexually transmitted. They argued that men could transmit it through sexual intercourse and it could go unnoticed because they present no symptoms.

*P1 – When someone has cervical cancer, if a man has intercourse with me when I have it, the man will pass it on to his wife, and if that woman also goes around then she will also transmit it, like this successively.*

*P2 – He doesn’t get ill*

*P1 – That man has it and goes around transmitting it to others [women].*

*P2 – It does not cause men pain...*

(Exchange between two participants during the FDG with female elders)

Discrepancies regarding the transmission of CC revolved around two conflicting conceptualizations. On the one hand, cancer itself was understood to be an illness that cannot be transmitted, whether sexually or not. On the other hand, CC could be understood as sexually transmissible because of where it was located (i.e. the uterus), as the following quote illustrates.

*P1 – Cervical cancer and cancer are different; if it [cancer] cannot be transmitted it means they are different.*

*[...]*

*P2 – Cervical cancer is transmissible because it is located near the genitals and they say it provokes discharge.*

(Exchange between two participants during the FDG with female elders)

In a similar vein, participants during the FGD with youth immediately identified the scenario case as an illness that was sexually transmitted, even though they did not refer to it as CC. They claimed that not washing properly after intercourse could lead to STIs. However, once they were told that was a case of CC, they retracted and claimed it was contracted differently. They understood cancers in general to be caused by the consumption of uncooked foods, which can cause internal wounds that lead to cancer.

Only the group of matrons understood CC to be hereditary because, to their knowledge, all cancers are. Like the group of youth, they also explained that it could be contracted through food, in this case expired food or food that has been grown with contaminated fertilizers. Lastly, adolescent girls understood CC to be transmitted through clothes, although they were unable to explain further.

## Discussion

To the best of our knowledge, this is one of the few qualitative studies to specifically examine lay knowledge of CC in SSA. There is a considerable amount of (mainly quantitative) studies that assess levels of awareness and knowledge of CC but often do so superficially, frequently limiting knowledge assessments to questionnaires that ask whether participants have heard of CC without further probing [31, 32]. This is partly due to the fact that most of these studies are primarily concerned with assessing HPV vaccine or CC screening acceptability and thus CC knowledge is treated as an addendum [14, 33–35].

If awareness is defined by the bare recognition of the term, this study finds that there is a relatively high level of awareness of CC among the study population. This correlates with the quantitative component of the study which found that 94% of the participants in Manhiça (in the case of the quantitative study these were exclusively adolescent girls) had heard of CC [29]. However, the qualitative component allowed us to delve deeper into the de facto knowledge of CC and so lead us to compatible yet different conclusions. In this case, the methodological advantage of the qualitative method allowed us to distinguish between recognition of the term through probing and spontaneous reference to it. Considering that only a few of the participants mentioned the term whilst brainstorming about known uterine ailments, it becomes apparent that this is not a term that most are familiar with. A study in the DRC found a similar discrepancy as only 12% of respondents mentioned CC spontaneously whilst 82% reported having heard about it when specifically inquired [36]. Studies that address this issue in SSA do not report consistent results since in some cases awareness appears to be high [37–40] and in others low [41–44].

In addition, our results suggest that participants were more familiar with the term *cancer* than with *cervical* cancer. This finding aligns with results of studies conducted in Kenya [45] and Tanzania [37]. In the latter study, only 1 out of the 37 teachers that participated had heard of *cervical* cancer, even though all had heard of cancer. The study in Kenya found that 91% of the 388 women surveyed had heard of cancer whilst only 29% had heard of *cervical* cancer.

With regards to knowledge of CC (signs/symptoms, aetiology), studies repeatedly conclude that it is very limited across locations, and this study is no exception [14]. Although most participants exhibited poor knowledge of CC, the few who were knowledgeable were considerably so, particularly with regards to how it manifests symptomatically. This is consistent with the findings from the quantitative component of the study, which concluded that participants identified abnormal vaginal bleeding (34%), vaginal discharge (40%) and dyspareunia (31%) as symptoms of CC [29]. Moreover, many studies from across the region have repeatedly concluded that in spite of limited knowledge, CC symptoms are easily recognized, even if not always associated with the biomedical term [46]. For example, a qualitative study with women from different regions in Nigeria found that most participants were able to identify some of the symptoms of CC, such as abnormal vaginal bleeding, offensive vaginal discharge and abdominal pain [16]. It is important to note that in our study the only ones that were able to describe in depth the symptoms and presentation of the illness were older adult women that had witnessed a relative experience the disease.

In order to gain more clarity regarding the diverse and layered nature of our study population’s knowledge of CC, we identified five levels that exhaustively represent this diversity: 1) those familiar with the biomedical term and holding a definition of CC compatible with a biomedical one, 2) those who had heard of the biomedical term but were not familiar with the disease manifestation or definition, 3) those familiar with the symptoms of CC as well as with the biomedical term but did not relate the two, 4) those familiar with the symptoms of CC but not aware of the biomedical term, therefore relating them to other diseases (mostly STIs) and 5) those not familiar with the biomedical term nor the symptoms of CC. Most participants belonged to the third and fourth groups.

Beyond these categories, our study allowed us to examine participants’ knowledge of CC in depth. We were thus able to identify (mis) conceptions about CC. On the one hand, there is the prevalent understanding of cancer as an illness that causes ‘wounds that don’t heal’. On the other, cancer is perceived to not take place in the cervix or the uterus because it is believed that the wounds it causes only happen in visible parts of the body, such as arms and legs. In this sense, the way cancer is defined is in line with biomedical knowledge, whilst the second conceptualisation does not allow CC to be defined in this way.

Moreover, the term *cancer* creates confusion when estimating whether CC is sexually transmissible precisely because cancer is deemed to be an affection of other more visible body parts that are not associated with sexual intercourse or reproduction. Due to this misconception, it is often assumed that CC cannot be sexually transmitted. Furthermore, the fact that cancers are generally not transmissible further complicates the notion that CC could be transmissible in any way, whether sexually or not. At the same time, the fact that CC (in Portuguese ‘cancro do colo do útero’ – loosely translated as ‘cancer at the entry of the uterus’) specifically refers to the uterus, a sexual and reproductive organ, made it easier for participants to associate it to sexual transmission, particularly as a consequence of contracting STIs. Although, as expected, HPV was never mentioned, the emic perspective makes a strong association between sexual practice, STIs and CC. In this sense, these findings are consistent with those of the quantitative component, which showed that 74% of participants considered sexual transmission as the mode of acquiring CC. Other studies also reported sexual connotations regarding CC, even when overall knowledge of CC was limited. A community survey in Uganda found that 85% of respondents (out of 454) believed that CC is sexually transmitted [47], whilst a qualitative study with women in Malawi found that half of participants considered STIs to increase the risk of developing CC [48].

To our knowledge, this is the first study to date that has revealed lay knowledge of the role of male circumcision in CC prevention. This heightened awareness of the relationship between male circumcision and STIs/CC can be understood when examining the health policy scenario in the Mozambican context during the years preceding the study. Since 2007, Mozambique implemented Voluntary Medical Male Circumcision (VMMC) programs using the operational guidance of the WHO and UNAIDS, which emphasized the importance of advocacy and communication strategies for successful program adoption [49]. By 2012, due to the 2-year funding program of the President’s Emergency Plan for AIDS Relief (PEPFAR), the scale-up process was speeded up, translating in an increase from 4009 VMMCs performed in 2010 to 68,924 in 2012 [50]. This process was accompanied by health campaigns to increase demand and acceptability, which lead to a nearly fourfold increase in acceptability from 2010 to 12 [50], just a year before this study begun. Thus, even though campaigns focused on HIV prevention, this health policy context sheds light on the origins of our study populations’ knowledge of male circumcision as an STI prevention method.

These findings show that, to a certain extent, lay perceptions of CC do align with biomedical ones. On the one hand, participants readily accepted CC as sexually transmissible due to the fact that it affects a reproductive organ and were very familiar with CC symptoms (even if unaware of the biomedical term). We also found a recurrent understanding that exposure to STIs could lead to CC. The fact that cancer in is conceptualized as an illness that causes ‘wounds that don’t heal’ is in line with the lesions that develop in the cervix as precursors of CC. Lastly, the notion that men can transmit CC without developing symptoms and the fact that male-circumcision can mitigate the transmission of CC, also aligns with biomedical knowledge [51, 52]. On the other hand, our findings also reveal discrepancies between lay perceptions and biomedical knowledge of CC. The notion that cancer is a condition that only takes place elsewhere in the body, such as in the breasts or limbs, and thus not in the cervix/uterus, contradicts biomedical knowledge. This is also the case regarding the perception that cancer is an illness that is mainly a concern in Western countries, as well as the notion that it is exclusively hereditary. Lastly, the fact that CC symptoms are compatible with other medical conditions (generally STIs), compounded with the lack of familiarity with the biomedical term, limits the ability to associate the two.

Health interventions should consider these findings if they aim to become contextually relevant and increase their efficacy. Health education campaigns often focus on efforts to improve lay people’s knowledge regarding the presentation of the disease. These campaigns should thus consider the gaps in knowledge and levels of awareness identified in this study, as well as address the misconceptions, when tailoring health messages to a given community. However, since CC is a chronic disease that only becomes symptomatic in terminal stages, increasing knowledge and recognition of its symptoms should not be a priority, as is the case with other, mostly acute, diseases. Thus, the focus should be on knowledge of risk practices and preventive actions (safe sexual practices, screening and vaccination). In the case of our study population, knowledge of symptoms and transmission of CC was generally limited. Yet, the ease with which the disease is associated with sexual transmission provides fertile ground for campaigns to focus on the preventive aspect of the disease and frame it from a sexual and reproductive health standpoint. Education messages and communication strategies should consider and address the misconceptions, as well as capitalize on the common ground, identified by this study. A shift of focus in information, communication, and education messages is required.

## Conclusions

This study has shown the extent to which lay knowledge of CC in southern Mozambique (the emic perspective) aligns with biomedical knowledge (the etic perspective). This has involved an in-depth analysis of the study population’s perceptions, conceptualizations and associations regarding CC, as well as a process of identifying points of convergence and divergence between both perspectives. Although knowledge of CC was generally found to be limited, some points of convergence were found, particularly regarding transmission. These findings confirm there is already a common ground to frame future health interventions from a sexual and reproductive health standpoint.

## Limitations

One of the main limitations of this study was the inability to recruit a higher number of participants in the unlicensed drug vendors group, which led to a focus group discussion being composed of a considerably lower number of participants than is recommended [6–12]. Researchers found it hard to recruit this group because, due to the nature of their work, they are fearful of repercussions and are thus averse to speaking openly about their experience, especially with researchers that are perceived to be related to the formal health system. In addition, discussions with adolescent girls were comparatively much less lively than with other target groups and so the data collected was poorer. This is likely due to shyness, which could in turn be due to the age difference between them and the facilitators and the latters’ inability to transmit sufficient ease. These aspects limited the contribution that these target groups made to the study.

## Data Availability

The datasets used and/or analysed during the current study are available from the corresponding author on reasonable request.

## Abbreviations

CC: Cervical Cancer
CHW: Community-based Health Workers
CISM: Manhiça Health Research Centre
FGD: Focus Group Discussion
HPV: Human Papilloma Virus
LMICs: Low and middle-income countries
MoH: Ministry of Health
NCD: Non-Communicable Disease
PEPFAR: President’s Emergency Plan for AIDS Relief
SSA: Sub-Saharan Africa
STI: Sexually Transmitted Infections
UNAIDS: Joint United Nations Programme on HIV/AIDS
VIA: Visual Inspection with Acetic Acid
VMMC: Voluntary Medical Male Circumcision
WHO: World Health Organisation
WRA: Women of Reproductive Age
